# Reablement professionals’ perspectives on client characteristics and factors associated with successful home-based reablement: a qualitative study

**DOI:** 10.1186/s12913-021-06625-8

**Published:** 2021-07-06

**Authors:** Mads Nibe Stausholm, Louise Pape-Haugaard, Ole Kristian Hejlesen, Pernille Heyckendorff Secher

**Affiliations:** Department of Health Science and Technology, Aalborg University, Aalborg, Denmark

**Keywords:** Home care services, Rehabilitation, Restorative care, Primary health care, Interprofessional relations

## Abstract

**Background:**

To understand what is needed to achieve a successful Danish home-based reablement service from the perspective of reablement professionals.

**Methods:**

Semi-structured interviews and observations were conducted with nine professionals within a municipal visitation unit in the Northern Denmark Region. Thematic analysis was used to analyze the interviews.

**Results:**

Four major themes emerged during this study: “*Heterogeneity of clients and mixed attitudes towards the reablement intervention*”, “*Shared understanding and acknowledging the need for help as the first step in reablement*”, “*Commitment and motivation are essential for successful reablement*”, and “*Homecare helpers as most important team players*”. The findings indicate that the clients had both mixed characteristics and attitudes about participating in the reablement intervention. Essential factors for successful reablement included a shared understanding of the reablement intervention, commitment, and motivation in terms of client involvement and staff group collaboration.

**Conclusions:**

Shared understanding of the reablement intervention, commitment, and motivation was found to be essential factors and the driving forces in relation to successful reablement.

**Supplementary Information:**

The online version contains supplementary material available at 10.1186/s12913-021-06625-8.

## Background

The population is aging worldwide, resulting in a growing proportion of dependent elderly people [1]. These demographic trends increases the incidence of older people experiencing decline in health and function, leading to more hospitalizations, higher healthcare service utilization, and thus increased health and social costs [1].

Traditionally, these trends have been met by greater use of homecare, where caregivers visit clients’ homes to provide personal care and perform activities of daily living (ADL) tasks over a long-term period. However, maintaining standards in the care sector becomes problematic with increasing workload and growing demand for care [2]. Consequently, there is a need for new healthcare services that can optimize independence and assist older people in improving their abilities to perform ADL [3, 4], and possibly an increase in workforce. In addition, many older people prefer to remain in their homes, creating an increased need for services that can support older people staying at home for as long as possible [5, 6].

Reablement (or restorative care) can be described as a service that “*aims to help people with disabilities recover their physical function through performing activities of daily living rather than having formal or informal caregivers perform these activities for them* [7, 8], *focusing on supporting independence*” [9]. Reablement differs from traditional home care, which involves personal care and cleaning for people with disabilities [7]. The following key elements are present in reablement: assistance by interdisciplinary teams; time-limited service for 6–12 weeks; a goal-oriented plan defined by the clients; outcomes in terms of improved ADL and health-related quality of life (HRQoL); and decreased service utilization [10].

Systematic reviews have synthesized the best available evidence of the effectiveness of time-limited reablement services. Several systematic reviews indicate that the effectiveness of reablement is promising in relation to a range of positive outcomes for reduced service utilization and for improved HRQoL and ADL [10–13]. A review by Sims-Gould et al. showed promising effects on the service usage and functional abilities of clients with evidence suggesting improved mobility and delayed deterioration [11]. In contrast, no positive effects on HRQoL were found [11]. Another review reported that reablement may improve functional status and decrease care costs to a small degree. However, the quality of evidence for these results was described as very low [14]. A review by Legg et al. concluded that reablement was an ill-defined intervention with no proven benefits in terms of increased personal independence or reduced use of home care services [15]. These two reviews [14, 15] suggested the need for more robust evidence of the effects of reablement and concluded that uncertainty exists regarding the size and importance of effects due to low-quality studies.

Scientific literature has also sought more qualitative research demonstrating how reablement can be structured and optimized to address the needs of clients and care providers [11, 16]. More research of who would benefit from reablement with respect to specific eligibility criteria is needed due to the heterogeneity of populations studied to date [10, 12, 14, 16]. Research concerning which components and processes of reablement are most beneficial, research into the role of occupational therapy, and input from other healthcare professionals are required [12–14, 17, 18]. In addition, there is a need for more process evaluation to assess professionals’ and clients’ views, experiences, and attitudes with reablement as well as which mechanisms and contexts can contribute most to the effects of reablement [14]. The knowledge base appears inconsistent, given limited evidence from existing randomized controlled trials and systematic reviews in the area [16]. However, practice-based evidence indicates that reablement is widely used and seems promising for people with functional disabilities [13].

In Denmark, the municipalities are the main actors in the field of rehabilitation and home care, and they are responsible for providing support and services to citizens. Since January 2015, Danish municipalities have been required to offer home-based reablement to adults with rehabilitation potential [19–21]. Reablement has been implemented in diverse ways, that, however, in line with the key elements of reablement [10], all must be time-limited, individually organized, based on the client’s own needs and resources, individualized in terms of setting goals in collaboration between client and the interdisciplinary team, and the municipality must provide assistance and support to the extent that it is necessary for the client to achieve his or her goal [19].

A home-based reablement service has been implemented in a Danish municipality in the Northern Denmark Region. The intervention is a part of the routine home care services provided and is offered to elderly and/or disabled citizens with rehabilitation potential who apply for practical assistance, personal care, aids, food service, or cleaning. The purpose of the intervention is to improve quality of life, help the client become more self-sufficient, and at the same time reduce the need for and expenses of municipal services. This is achieved by focusing on active training instead of passive care, where the client’s own goals for significant activities are central and based on their motivation, resources, and needs. The individualized rehabilitation-based goals determine which efforts are to be initiated and professionals are to be involved. This can include, for example, aids, training, home modification, counseling, or something completely different. When an interdisciplinary reablement course is commenced, it involves reablement professionals (self-help coordinators, licensed therapists, self-help instructors or self-help therapists) from a municipal visitation unit and home care providers. The intervention is tailored to each client’s characteristics and needs. The reablement professionals and the client collaborate to identify initiatives and goals that must be established to support the client in the best possible way.

Existing research into how reablement operates in practice, which service components work best, who can benefit from the service, and clients’ reactions to reablement is sparse [10, 16]. Few empirical studies have already explored care professionals’ and clients’ experiences of reablement [22–37]. However, reablement services differ in both content and organization, making it difficult to compare existing research in the field. Moreover, it is relevant to consider both clients’ and care professionals’ perspectives when addressing these knowledge gaps in reablement studies. The Social Care Institute for Excellence (SCIE) have argued that there is no single definition of “successful reablement”, which may look different for different people depending on people’s abilities at the start of the service and other variables which include the motivation for progress and desire for setting goals [38]. Since there is no single universal definition of successful reablement, the present study focused on reablement professionals’ perspectives on client characteristics and the most important factors associated with successful reablement. Accordingly, the aim of this study was to understand what is needed to achieve a successful Danish home-based reablement service from the perspective of the reablement professionals. To address this aim, an understanding of client characteristics and successful reablement is needed; therefore, the following two research questions (RQ1, RQ2) have been identified:

- RQ1: How do reablement professionals describe clients participating in home-based reablement?
- RQ2: Which factors are associated with successful reablement from the reablement professionals’ point of view?

## Methods

### Research design

The study was designed as a thematic analysis with a qualitative hermeneutic phenomenological approach to generate empirical knowledge of reablement professionals’ perspectives on client characteristics and success factors of a home-based reablement course [39, 40]. This research design was chosen, as reablement professionals who have first-hand experience of reablement can characterize the phenomenon as they experience it and recount their perspectives on client characteristics and success factors.

### Research setting

The research setting was a municipal visitation unit located in a Danish municipality in the Northern Denmark Region. The visitation unit was further divided into two subunits that either carried out short-term or long-term rehabilitation efforts, organized according to clients’ needs. The key features of the reablement intervention are described in Table 1.

**Table 1:** Key features of home-based reablement service

Client request • An assessment of the client’s situation and rehabilitation potential is undertaken
**Meeting between self-help coordinator and client is arranged to clarify**: • The client’s need for help • Whether or not it is possible to rehabilitate the client o If yes: Individualized goals, timeframe, and content for rehabilitation are agreed o If no: Compensatory help is initiated
**Coordination of the reablement course** • The reablement course is coordinated with relevant actors, such as the training unit, homecare providers, nursing home care providers, food service staff, etc.
**The individualized reablement efforts are initiated based on the client’s needs and consist of**: • Assisted training with self-help instructor/self-help therapists in daily activities such as dressing, bathing, preparing and eating food, cleaning, vacuuming, or visiting health centers, etc. • Repetitive training with home care providers who typically know the clients in advance
**Reablement – follow-up** • The self-help coordinator follows up on the reablement course and re-considers the client’s rehabilitation potential
**Reablement – status** • New reablement efforts are initiated or the reablement course ends

### Study participants

The informants were identified by heads of the visitation unit and were selected using convenience sampling. The informants (*n* = 9) were reablement professionals with specific functions in the reablement service. All informants were women aged 25–61 (mean = 41) and had 1–6 years (mean = 3) of experience with the reablement intervention. The reablement professionals included self-help coordinators (*n* = 4), a self-help instructor (*n* = 1), self-help therapists (*n* = 2), and licensed therapists (*n* = 2), all with healthcare backgrounds as nurses (*n* = 1), physiotherapists (*n* = 1), or occupational therapists (*n* = 7). The self-help instructor and self-help therapists carried out the same work tasks, but they were named after which of the two subunits they were employed. Table 2 describes the specific roles of the informants.

**Table 2:** Reablement professionals’ roles in reablement

Occupation	Role
Self-help coordinator	• Assessing the client’s rehabilitation potential • Setting individual goals in cooperation with the client • Coordinating efforts and finishing reablement courses
Licensed therapists	• Granting of aids
Self-help instructor/self-help therapists	• Individual training with the clients • Educating and involving homecare providers

### Data collection

Data were collected between March and April 2017. Semi-structured interviews (*n* = 9, duration = 37–60 min) and observations (*n* = 7, duration = 45–60 min) were conducted with reablement professionals. The interviews took place in the visitation unit in a quiet room with no one else present to avoid interruptions. The observations were carried out in the clients’ homes.

The informants were invited by email to which participant information was attached that explained the study’s purpose and the researchers’ goals. The informants received written and oral information and gave their consent before the interviews to build trust and reduce nervousness. The semi-structured interviews were the main source of empirical data and offered in-depth information on the explored phenomenon. Observations took place immediately before each interview to help qualify the interviews and obtain a pre-understanding of the reablement concept, and professionals’ workflow and interactions with the clients in relation to the reablement intervention. During observations, quick memos were made using key words. The researchers split up during the observations due to professionals’ concerns for the clients who could feel discomfort by being observed and to affect the research setting as little as possible. The interviews were conducted in Danish by the same two researchers (PHS, MNS) according to an interview guide that aimed to address the research questions. Examples of interview questions were: “*How does the client typically react to the reablement intervention?”* and “*How would you describe a successful reablement intervention?”.* The interview guide was developed specifically for this study (see Additional file 1 for further details).

The researchers were free to vary the wording and order of questions during the interviews. The interviews were audiotaped, and quotes that appear in the paper were freely translated into English.

### Data analysis

Transcripts corresponding to one hundred pages were coded independently by both researchers based on a common codebook developed through reading the transcripts. The common codebook was utilized to improve reliability by creating a structure and agreement about definitions of codes and themes [41]. Labels for codes were derived from data during the data analysis and became the initial coding scheme. The codebook consisted of 104 codes divided into 14 main codes and 90 sub-codes sorted based on their relationship. The interviews were transcribed verbatim, and all transcripts were validated by both researchers by analyzing both separately and together to obtain a first impression of the content of the interviews and a deeper understanding of the data. Data saturation was considered achieved when no emergent themes or codes were identified by the researchers. A thematic analysis using an abductive approach was performed using Nvivo11-software [42]. To assess inter-rater agreement, both percent agreement and Cohen’s unweighted Kappa were calculated [43]. Cohen’s Kappa was calculated individually for each combination of source and node. Member checking, stepwise replication, and external audit were used as techniques to establish credibility and dependability [44]. The thematic analysis was based on the following steps:

- The interview recordings were split between the researchers and full transcripts were produced.
- The researchers read each other’s transcripts while listening to the corresponding audio-recording to validate and acquire a first impression of the content.
- The researchers read all transcripts to identify codes separately.
- A common codebook was developed based on all of the identified codes that the researchers had found separately.
- The transcripts were transferred into Nvivo11, where the researchers coded separately according to the common codebook.
- The researchers made generalized descriptions regarding the reablement professionals’ perspectives on client characteristics and success factors of the reablement intervention.
- The researchers discussed the interpretation of the findings until agreement was reached.

The COREQ (consolidated criteria for reporting qualitative research) checklist was consulted for reporting the results of the manuscript (see Additional file 2 for further details) [45].

## Results

Inter-rater reliability was assessed by calculating percent agreement and Cohen’s unweighted Kappa [46]. The percent agreement was 98.85 % and Cohen’s Kappa was 0.40, indicating weak [47], fair [48–50], or fair-to-moderate agreement between the coders [51]. While answering RQ1 and RQ2, the following themes emerged: “*Heterogeneity of clients and mixed attitudes towards the reablement intervention*”, “*Shared understanding and acknowledging the need for help as the first step in reablement*”, *Commitment and motivation are essential* for successful reablement, *and “Homecare helpers as most important team players”*.

### Heterogeneity of clients and mixed attitudes towards the reablement intervention

 All informants agreed that there, in general, were no clear definition of clients who were referred to reablement and that client characteristics may vary even for clients with similar anticipated rehabilitation potential. The clients had different life situations and could be new to or familiar within the municipal system. No stereotypes existed, but most of them were elderly. One informant claimed:

No, there are no stereotypes, but mostly older clients are represented. There are also many younger ones. When I say older, I mean 65 years or more. It might even be 79–80 years or more.

On the other hand, the informants stated that there seemed to be similarities between clients suffering from the same disease, such as chronic obstructive pulmonary disease, cancer, or arthritis, as illustrated in this quote:

But of course, clients suffering from COPD often have an anxiety problem due to breathing trouble.

The informants also reported that clients had mixed attitudes towards the reablement intervention. Several of the informants highlighted the clients’ understanding or lack thereof, desired independence, and motivation as typical reactions to the reablement intervention.

### Shared understanding and acknowledging the need for help as the first step in reablement

Understanding was related to the clients’ acceptance of being screened for rehabilitation potential and possibly undergoing a rehabilitation course to receive help from the municipality. Two informants stated:

Well, that is something they have to go through to receive help. They know it! To receive help from the municipality, they must be screened for rehabilitation potential.

Those we have here in the section for long-term care in the visitation unit usually come from the section for short-term care, and, to begin with, they must go through the same procedures when they enter the municipal system. They have already completed a reablement intervention or similar and know that when new problems come up, the municipality must assess whether the client can manage the task or not.

From the point of view of the informants, understanding reablement was also linked to the clients’ recognition of a given problem, as illustrated in this quotation:

If the client experiences a problem and would like to get better, they react in a way where reablement is considered a help.

The informants described the importance of helping the clients to acknowledge their need for help. Reablement was then considered a meaningful and helpful service for the clients.

Conversely, lack of understanding was related to lack of recognition of having a problem. It may be difficult to acknowledge the need for help, for example, from aids, as it is a very visible sign of impaired functioning. This lack of recognition was exemplified in the following quotation:

I cannot force them, they must be prepared for it, but I think it can be difficult for people to accept that they need an aid. It´s often in people’s own minds, because you can also say that it is visible to others that they really need help, but they do not themselves realize they need it.

Lack of understanding was also expressed when old age or habits were not reason enough to be provided help if the need for such did not exist. One informant expressed this point as follows:

Yes, some people think that it’s absurd – “why cannot I [the client] get help getting washed on my back?” or “It’s just nice that you wash my hair” or “I cannot understand that I have to do it myself, now that I am 85 [years old]!”

They [the clients] have paid taxes throughout their lives, and this makes it hard to understand that we come and want them to do it [participate in reablement/themselves].

Another typical reaction was the desire for independence – especially regarding personal care. The majority would like to take care of themselves. By contrast, informants described that clients were not always satisfied with theamount of practical assistance, such as cleaning:

Many of them [the clients] want independence – others like to get daily visits where practical assistance [e.g. cleaning or bathing] is included. This may create a dilemma in cases where the municipality wants the client to be independent rather than it being the client’s own desire.

The informants expressed that some clients preferred getting help because they did not have the strength or the interest to clean their home. In that case, more resistance from the clients was experienced. The informants stated that clients could find it strange to be checked and trained in something they could manage but did not want to do anymore. Therefore, the informants identified the importance of moving the clients’ focus from passive to more active roles in reablement.

### Commitment and motivation are essential for successful reablement

The informants stated that it was vital that clients as well as actors such as home care providers understood the purpose of the reablement intervention. One informant mentioned that the best results were obtained when all involved actors participated with great commitment. Another informant described the quality of information given to clients as very important, as illustrated by the quote:

I think that it’s the feeling I have while visiting a client. That it is going well, and you get to talk, and you feel that the client is informed, and I get the information I need. Having informed the client so that he or she knows what is going to happen in the future and feels safe.

From the informants’ perspective, a central aspect of reablement is empowering clients and making them as self-reliant as possible with or without the use of aids. The informants made the point that some clients had received services prior to participating in reablement, where the goal of the reablement intervention, depending on the client’s situation, was to maintain or improve the current level of functioning, and to reduce or maintain the current need for help. An alternative could be supporting the clients in clarifying what they were not able to accomplish and find other ways of managing the problem. Getting the client to acknowledge his or her limitations, as well as identifying other solutions for doing activities was a major part of reablement. Thus, the informants felt responsible for motivating clients to gain more self-control and to make them understand that they were not necessarily hampered by their perceived level of functional capability. The informants highlighted that empowerment was an important factor in reablement.

One informant felt that a successful reablement course included giving clients experiences of success and satisfaction. The informants expressed the importance of setting individual goals in collaboration with the client and identifying their need for support, and that finding possible solutions was an important part of their work. In addition, the informants described the relevance of getting people to live their lives again by supporting networking where enhanced quality of life as an outcome is an important factor in the reablement intervention, as illustrated by the quotation:

For various reasons, the home care providers are the primary social network in the reablement courses that I have had. Clients insist on keeping their municipal services. But we can actually support clients in getting a better social network and continuing networking, and we stay with them until it´s established. The purpose is to make them independent of passive services and become as self-reliant as possible, and I think it affects HRQoL.

Several of the informants highlighted motivation as being of major importance to the reablement intervention. Motivation was expressed in different ways. One informant said:

It is also the healthiest [of clients] who get the most out of it and are motivated.

The informants described the importance of the clients’ success experiences in the initial stage of reablement as they were closely related to enhanced motivation. The informants also mentioned chemistry between clients and the reablement team as important for enhancing motivation. A tendency of enhanced motivation existed when professionals could prove to the clients that their role was to support them in the best possible way to become fully or partially self-reliant.

All informants reported that motivation in terms of user involvement and staff group collaboration was an essential aspect of a successful home-based reablement intervention.

The informants stated that some clients were already motivated in the initial stage of reablement, while others might become motivated over time. However, motivation was closely linked to the understanding of reablement and individual goal setting. The reablement intervention should make sense before clients were motivated to continue. Several informants pointed out that they therefore prioritized motivating and informing clients adequately before start-up:

Motivation is our keyword, always. That’s what we do most since we have to motivate everyone on our way.

To promote motivation, the informants expressed the importance of setting individual goals for each reablement course in close collaboration with the clients. Motivation could also arise during the reablement intervention when clients experienced progress through training. Success experiences with the training resulted in enhanced motivation and trust between the clients and reablement professionals.

Motivation was also necessary in relation to collaboration between the reablement team and other staff groups. The informants highlighted the importance of shared understanding between the reablement team and other involved actors. They emphasized that they had the main responsibility for carrying out reablement and were the link between many parties, and therefore had to motivate and identify where to act first with their collaborators. The informants agreed that motivation was the driving force behind reablement interventions.

### Homecare helpers as most important team players

All informants mentioned the home care provider as the most important collaborator because home care helpers play an important role in reablement by making observations and training with clients. The informants emphasized home care helpers as the largest employee group, and home care helpers must be prepared to carry out reablement in practice. Home care helpers were typically also closest to the clients and mutual trust was already established between them. Initially, this trust was often not present between the clients and reablement professionals. One informant stated:

Most clients keep up a façade when a reablement professional arrives, but do not necessarily keep up that façade with their regular home care helpers.

The informants described the relationship between home care helpers and clients as beneficial as the home care helpers’ regular visits contributed to them knowing the clients’ needs. Therefore, the informants felt a stronger collaboration when the home care providers were involved early in the process – typically during the first visit. The informants mentioned that during the first visit, it is possible to observe the home care helper’s interaction with the client. Based on these observations, the client, home care provider, and reablement professionals jointly discuss the client’s goals and the specific content of the reablement intervention. Subsequently, the home care helper receives instructions from the reablement professional on how to train with the client in his or her own home during routine visits.

Motivation among home care helpers could also be challenging due to limited time resources. The informants highlighted that some home care helpers considered reablement to be an extra workload. The older and most experienced home care helpers in particular could get stuck in old habits and be critical of reablement. This critical attitude could lead to lack of motivation, possibly affecting some clients negatively.

However, the informants mentioned that a paradigm shift was slowly happening, with home care providers changing their mindset from providing direct physical care to providing more rehabilitative care as illustrated by the following quotations:

I think it is getting better and better! The more it gets rooted. It reflects a paradigm shift in home care. They change their mindset from thinking of themselves as “warm hands” to rehabilitating employees.

I can feel that there is a difference, and people are beginning to understand the rehabilitative approach or thinking that is involved. I think we are getting closer to a common mindset, but it has taken a long time.

## Discussion

### Main findings

This study aimed to explore reablement professionals’ perspectives on client characteristics and factors associated with a successful home-based reablement intervention. Interviews were conducted with reablement professionals, and a thematic analysis was applied. The findings of this study led to four major themes being identified: *“Heterogeneity of clients and mixed attitudes towards the reablement intervention”, “Shared understanding and acknowledging the need for help as the first step in reablement”, “Commitment and motivation are essential for successful reablement”, and “Homecare helpers as most important team players*”. The findings indicate that the clients had both mixed characteristics and attitudes about participating in the reablement intervention. The informants reported client- and staff-related factors that were considered to be associated with successful reablement. Amongst others, these included shared understanding of the reablement intervention and overall, commitment, and motivation was essential factors and the driving forces in relation to successful reablement. Minor themes were also identified but not included due to low frequency of codes amongst informants.

### Discussion of findings

 There was broad agreement among informants that clients differed so much that they had only very few characteristics in common. They all possessed rehabilitation potential as they otherwise would not have been included in the reablement intervention. Only one informant stated that clients with the greatest resources also benefitted the most from the service. This is in line with the study by Rabiee et al. [30] which suggested that outcomes are likely to be lower for clients with more limited potential to be independent and are less likely to be effective for those who need ongoing support. However, only one informant in our study shared this sentiment, which suggests uncertainty about which population groups might benefit the most from using reablement services. Reablement may be more effective for certain client groups, and further research is required to investigate this topic, as confirmed by Tessier et al. [10] and Aspinal et al. [16] However, it might also be considered that those clients with most resources and high motivation might benefit irrespective from the reablement service. Furthermore, it can also be assumed that clients with poorer health and motivation can benefit most from the intervention.

In addition, the informants mentioned that the clients had diverse reactions to the reablement intervention. The clients reacted with motivation, understanding, and a desire for independence or lack thereof. Understanding was generally related to the screening process and recognizing the need for assistance. To ensure understanding, reablement professionals played an important role in communicating the aim and content of the reablement intervention in an understandable way during the initial phase of the reablement course. Jokstad et al. [52] have explored strategies to facilitate clients’ understanding by spending sufficient time, having patience during the initial stage of a reablement intervention, and introducing small tasks that clients can manage. Moe et al. [36] confirmed the importance of professionals’ communication skills in the initial phase of the reablement course as they promote client influence and shared responsibility in developing clients’ goals.

Enhanced motivation was linked to experiences of success with the reablement intervention and mutual trust between professionals and clients. The link between motivation and successful reablement is supported by Rabiee et al. [30] who found that clients’ motivation influenced the effectiveness of reablement. The informants highlighted their central role in changing the clients’ focus from being passive users to being active users of reablement and creating success experiences in collaboration with the clients. The informants stated that changing the clients’ focus could be particularly challenging due to the passive user role that clients usually take. This is in line with Jokstad et al. [52] who argued that some users do not take control and demonstrate the active user involvement that health professionals have as an ideal. This is supported by Holm et al. [53] who suggested that the transformation from passive to active users is challenged by traditions where users remain in a passive patient role.

Important factors associated with successful reablement were shared understanding, commitment and motivation from all involved actors in reablement. The informants felt responsible for promoting these success factors through increased information and cooperation with clients and collaborators. The informants’ described the following success factors: supporting clients’ goals, social networking, and being motivated. This is in line with Moe et al. who argued that successful reablement depends on effective mapping processes and professionals’ excellent communication skills to promote goal-setting [36]. Goal-setting may lead to better insights into clients’ everyday lives and may improve their participation in the reablement process [54, 55].

The informants highlighted the importance of promoting motivation by involving clients in setting individual goals for the reablement intervention. Similar findings were presented in Randström, Hjelle and Birkeland [31, 34, 37] who described the importance of enhancing motivation by drawing attention to users´ needs and supporting them in defining their own goals. This is also supported by Latham [56] who argued that goal setting facilitates motivational benefits, which supports the importance of enhancing clients’ motivation and performance through goal setting.

The informants emphasized the importance of supporting clients’ networking to prevent loneliness. Elderly can be lonely which can influence the clients’ willingness to participate in reablement. While the fear of loneliness may increase motivation for reablement, it might also be assumed that clients who are lonely may be less motivated to become independent and thereby hold on the help. This is supported by Valtorta and Hanratty [57] who argued that dealing with loneliness amongst the elderly will reduce health inequalities and enhance individuals’ quality of life. Furthermore, depression and cognitive decline might also influence the willingness or ability to participate.

The informants stated that motivational work was deemed the driving force for successful reablement intervention. Similar findings were presented by Hjelle [37] who found that driving forces in the reablement process resided in dynamic interaction between intrinsic and extrinsic motivational factors. In general, motivation was considered a precondition for user involvement and staff collaboration; in this regard, the reablement professionals´ interpersonal qualities and communication skills were essential. The informants described how motivation varied among involved actors in reablement, e.g. for home care helpers and clients. The clients’ level of motivation was typically related to their understanding of the reablement concept and their experiences of success with training activities.

The informants’ description of client-related success factors included enhancing their self-reliance, self-control, independence, quality of life, satisfaction, and motivation. Client-centered outcome measures are vital when evaluating reablement services, and studies have highlighted the importance of exploring users’ own views and perspectives on rehabilitation [37, 58, 59].

Motivation was also essential when collaborating with other staff groups, especially home care providers, as they play a key role in training with the client during the reablement intervention, especially if trust between the home care helper and the client had already been established. Therefore, motivation and shared understanding were needed for optimal interdisciplinary collaboration. At times, the informants experienced resistance from home care helpers who claimed that the reablement intervention was time-consuming and represented additional workload. In addition, several home care helpers had difficulty understanding and accepting the mindset of reablement. This is supported by Hjelle et al. [35] who stated that effective reablement requires a shift in work culture from a static to a dynamic service, which is time-consuming, and a better framework to support the adaptation of the reablement approach to thinking and working. In our study, the mindset of the home care providers was challenging but they gradually shifted from thinking of their work roles as passive carers to becoming more active rehabilitating employees. As such, reablement professionals had an important role to play in transforming the home care providers’ mindset by attaching higher priority to communication and motivational interviewing. No standard protocols or tools were used to motivate either clients or staff and some conditions may have challenged the provision of reablement, which highlights the importance of standardized tools that can support the provision of reablement.

### Methodological considerations

#### Strengths

A qualitative approach was applied to explore the reablement professionals’ perspectives on client characteristics and factors associated with successful reablement and to create valuable practice-specific insights. The informants represented a mixture of work functions, which provided several perspectives on the topic of interest.

Individual interviews were chosen rather than focus groups interviews, as the focus was on gaining detailed knowledge of the individual informant’s perspectives on the phenomenon. The majority of the informants have educational backgrounds as occupational therapists, which may limit the heterogeneity of perspectives. However, since reablement and supporting management of activities of daily living are core aspects of occupational practice this is considered a strength [60]. A common interview guide and a codebook were developed and contributed to the objectivity and trustworthiness of our study, making the findings more plausible [61]. Observations made it possible to minimize respondent-induced courtesy bias, where respondents tend to give the answers they think researchers want to hear, rather than truthful accounts [41]. A brief summary of the findings was presented through member checking with the reablement professionals to optimize validity and further reflect on informants’ experiences [62].

Two researchers with different professions and perspectives carried out the data collection and data analysis to improve credibility and dependability [63]. Two external researchers reviewed the processes of data collection and data analysis to ensure the dependability and consistency of the study’s findings [64].

#### Limitations

Given the small sample size and the gender imbalance, the findings from this study may affect the external validity and might not be representative of other health professionals working with reablement services. However, the gender imbalance may be difficult to avoid as the majority of health care professionals are females, which is also the case in Hjelle et al. [35] In addition, the participants’ perspectives cannot be generalized to other healthcare professionals’ working with reablement services as the municipalities in Denmark offer their own variant of reablement services. However, we believe that our findings can be used as inspiration in other reablement settings.

In connection with the publication, informants’ quotations were translated from Danish into English. Translating quotations into another language can be a delicate process that risks meaning loss. However, the translations were thoroughly discussed among the researchers and reviewed by a proofreader to increase the validity of the qualitative study [65].

The leaders of the visitation unit recruited reablement professionals for our study, and they might have been selective in their choice of informants, which may have influenced our findings. However, the leaders were not present during the interviews, and informants’ reflections were not only positive as different opinions existed and challenges were addressed. Furthermore, the informants were promised full anonymity.

Percent agreement and Cohen’s unweighted Kappa coefficient were calculated as a measure of inter-rater reliability to ensure consistency and trustworthiness of the two researchers’ coding. Percent agreement was 98.85 %, indicating a very high level of agreement that lies well above the minimum acceptable level of agreement proposed by McHugh. [47] However, the percent agreement does not take into account whether the level of agreement is due to chance or not, wherefore Cohen’s Kappa was evaluated [47, 66]. Cohen’s unweighted Kappa was 0.40, which indicates weak [47], fair [48–50, 67], or fair-to-moderate agreement between the coders [51]. The large difference between the percent agreement and the Kappa value may be due to the fact that the Kappa statistic corrects for the amount of chance rater agreement, while the percent agreement does not [47, 66]. A large degree of chance rater agreement may be attributed to the fact that large parts of the transcripts were not coded, which would lead to an expected level of chance agreement. According to McHugh [47], the greater the expected level of chance agreement, the lower the resulting Kappa value. This may help to explain the relatively low Kappa value.

In the case of imperfect agreement there is a substantial risk of reporting misleading findings, however, considering the nature and amount of the coded material and the very large number of codes, the inter-rater agreement level may be considered acceptable.

## Conclusions

The findings of this study emphasize the importance of shared understanding of the reablement intervention, commitment, and motivation in relation to successful reablement. Our findings have practical significance for stakeholders and health professionals, as they contribute to an understanding of reablement professionals’ perspectives on client characteristics and reactions to reablement; moreover, they help us understand factors that may lead to the success of reablement interventions. The insights of this study may contribute to improving reablement in practice. This study suggests that:

- Mixed attitudes but no similarities exist among clients with rehabilitation potential. Exploring which population groups might benefit more from reablement than others will contribute to a more person-centred approach and greater effectiveness of reablement.
- Common success factors among professionals and clients included shared understanding and commitment. In order to improve these success factors, a competence boost in home care helpers and more involvement of home care helpers as central players in reablement are recommended.
- Motivation was essential to client involvement, staff collaboration, and the success of the reablement service. The findings from our study suggest the importance of greater focus on supporting municipal healthcare professionals’ competences for motivating clients and other actors in reablement by use of standardized tools. Since no operational definitions of motivation were incorporated into practice, it is recommended to develop and adopt these.

Further research that includes the clients’ perception of the reablement intervention is needed to provide deeper understanding of who will benefit from reablement. In addition, research concerning what motivates reablement recipients and how reablement professionals can help build and maintain motivation may lead to the insights necessary to improve the effect of reablement interventions.

## Supplementary Information

**Additional file 1.** The interview guide.

**Additional file 2.** The COREQ checklist.

## Data Availability

The data generated and analysed during the current study are not publicly available due to the nature of the study sample and the informants’ feedback. The informants can easily be recognized and to avoid breaking confidentiality, data are available from the corresponding author on reasonable request.

## Abbreviations

ADL: Activities of daily living
HRQoL: Health-related quality of life
RQ1: Research question 1
RQ2: Research question 2
