# Supplementary material for: Reablement professionals’ perspectives on client characteristics and factors associated with successful home-based reablement: a qualitative study
Source: BMC Health Serv Res. 2021 Jul 6;21:665. doi: 10.1186/s12913-021-06625-8 (PMC8262059; doi:10.1186/s12913-021-06625-8)
Supplement: Supplementary file 1 — Additional file 1. The interview guide. [file 12913_2021_6625_MOESM1_ESM.docx]

| **Problem statement** | *How does a typical reablement course take place and how are the patient-related effects evaluated?* | |
| --- | --- | --- |
| **Theme** | **Research questions** | **Interview questions** |
| Descriptive/clarifying | How is the visitation unit structured in terms of reablement? | Can you describe the workflow with reablement as a whole (job roles/job titles)? |
|  | Which actors are involved in reablement?  - Internally/externally | Whom do you work together with regarding the individual client? (both internally and externally) |
|  | How large a proportion of the municipality's clients receive reablement? | How many clients receive reablement?  Is there potential to increase the number? |
|  | What are the self-help coordinators’/therapists’/instructors' perceptions of the purpose of reablement? | What is the purpose of reablement from your point of view? |
|  | What roles do self-help coordinators/  therapists/instructors have in reablement? | What is your job role in:  - the visitation unit  - reablement |
|  | How have the work roles/division of work changed through the development of reablement? | Has your work role changed since the reablement concept was introduced?  - More than once?  - How? |
|  | How are the competencies distributed around the different roles and work areas? | What competencies are required in your position/role?  Are there others with the same job title who possess some completely different competencies?  - Do they handle the role/task in the same style as you? |
|  | How does a client case proceed in reablement?  - Visitation before/after | When do you first hear about the client?  How and how often is a client referred for reablement/trained in reablement?  Describe a reablement case from your angle?  Are you outreach in relation to citizens with reablement potential? |
|  | Describe the typical client who is included in reablement | Is there a stereotype of clients in reablement?  Is there an equal distribution between clients who are already in the system and clients who are not yet in the system? |
|  | How are clients stratified to reablement? | What types of clients are included in a reablement course?  Which are excluded from a reablement course?  What tools are used for the visitation? |
| Collaboration | How do the employees from the visitation unit collaborate with external actors such as the client's family / doctor / etc.? | Do you have contact with other actors than from the visitation unit regarding the individual client?  - Who and how? |
|  | How do the actors collaborate internally regarding the individual client case? | How do you collaborate with the individual actors about the client?  Do you pass on the client to other actors when your work function is finished?  - Who and how? |
| Documentation | What IT systems are used for documentation? | What programs do you use to document/register information in relation to reablement?  Do you have any documentation requirements?  - How? |
|  | How often is it documented and what guidelines exist for this? | What do you need to document in relation to your work function and role?  What is documented in connection with the visitation?  How often do you have to provide documentation for the individual client?  Are there any guidelines that need to be adhered to?  What is recorded in the final documentation? |
|  | How do clients react to the reablement concept? | Do all clients react equally to the reablement process?  - Mentally?  - Physical/reablement?  Is the client's reaction documented in some places?  What happens in the situation where the client does not show potential to become more self-reliant?  - How is this reablement potential assessment made? |
| Evaluation of reablement | How is reablement and its effects evaluated? | Do you use the documentation for anything?  - Do you know if others use it?  Is it evaluated along the way or is it exclusively after the reablement course? |
|  | How does a successful reablement course develop? | What success criteria do you have in your work role/work function?  Have you set these up yourself or have they been set by others?  Are they comprehensive for your work effort? |
| Follow-up after reablement | What is the status of the client after completion of the concept? | Is the client's condition monitored for a period after the end of the reablement course?  - What is your opinion on this? |
| The client in reablement | How does the client act in the reablement concept? | What is the role of the client in reablement? (passive / active)  - Can the client be further involved?  How does the client react to the concept (in different situations)?  - How are the different reactions handled? |
| Advantages/disadvantages of the reablement concept | How does the reablement concept work purely conceptually? | Are there any pitfalls/limitations in reablement?  Would you do something different?  If you could define the following yourself, what would it be like?  - Your role in reablement?  - Reablement as a whole/concept? |
|  | How can the advantages be exploited to a greater extent and the disadvantages minimized? | Do you have suggestions on how to improve reablement? |
| Final comments |  | Do you have any special wishes/ideas for the reablement concept that have not been considered before?  Is there anything you would like to add at the end? |
